# Breast-lesions characterization using Quantitative Ultrasound features of peritumoral tissue

**DOI:** 10.1038/s41598-019-44376-z

**Published:** 2019-05-28

**Authors:** Ziemowit Klimonda, Piotr Karwat, Katarzyna Dobruch-Sobczak, Hanna Piotrzkowska-Wróblewska, Jerzy Litniewski

**Affiliations:** 1 Institute of Fundamental Technological Research, Department of Ultrasound, Pawińskiego 5b, 02-106 Warsaw, Poland; 2 Maria Skłodowska-Curie Memorial Cancer Centre and Institute of Oncology, Wawelska 15b, 02-034 Warsaw, Poland

**Keywords:** Diagnostic markers, Breast cancer, Cancer imaging, Biomedical engineering

## Abstract

The presented studies evaluate for the first time the efficiency of tumour classification based on the quantitative analysis of ultrasound data originating from the tissue surrounding the tumour. 116 patients took part in the study after qualifying for biopsy due to suspicious breast changes. The RF signals collected from the tumour and tumour-surroundings were processed to determine quantitative measures consisting of Nakagami distribution shape parameter, entropy, and texture parameters. The utility of parameters for the classification of benign and malignant lesions was assessed in relation to the results of histopathology. The best multi-parametric classifier reached an AUC of 0.92 and of 0.83 for outer and intra-tumour data, respectively. A classifier composed of two types of parameters, parameters based on signals scattered in the tumour and in the surrounding tissue, allowed the classification of breast changes with sensitivity of 93%, specificity of 88%, and AUC of 0.94. Among the 4095 multi-parameter classifiers tested, only in eight cases the result of classification based on data from the surrounding tumour tissue was worse than when using tumour data. The presented results indicate the high usefulness of QUS analysis of echoes from the tissue surrounding the tumour in the classification of breast lesions.

## Introduction

Breast cancer is still one of the leading causes of cancer-related death for women worldwide^1^. Methods are being sought that allow early and accurate diagnosis of cancer, and to precisely differentiate the character of the lesion and qualify for biopsy. X-ray mammography has reduced breast cancer mortality up to 45%, and is recommended as a screening method^2^, but its sensitivity is limited in women with dense breasts. In a group of patients with extremely dense breasts it is reported to be 48%^3^. Conventional ultrasound (US) is a complementary examination in women with dense breasts. It allows assessment of the cancer features according to the BI-RADS lexicon^4^, being faster, cheaper, and more accurate than mammography^5,6^. On the other hand, US has limitations such as reproducibility and quality of the equipment, and therefore is operator and device-dependent^7,8^. It is worth noting the great development of machine learning techniques in the field of breast cancer classification. Methods based on B-mode image analysis can achieve very high diagnostic performance^9–13^. A systematic review of the efficacy of cancer classification using various methods of machine learning and imaging diagnostics, including ultrasound, can be found in Yassin *et al*.^14^.

Evaluation of tumors based on the BI-RADS classification can be improved by including quantitative ultrasound (QUS) methods. While the B-mode ultrasound does not provide reliable quantitative information on the tissues examined, QUS techniques allow for the evaluation of tissue in terms of its structure and mechanical properties. They can be a valuable complement to ratings based on BI-RADS. Some QUS parameters are closely related to BI-RADS descriptors, while others go beyond the tumor traits described there^15^. The combination of QUS methods and the BI-RADS assessment has the potential to better identify patients with benign lesions and at the same time increases the accuracy of breast lesion classification^16^. The use of quantitative parameters enables clinicians to limit the number of biopsies, while still maintaining the same level of detection in terms of malignant cases^17^. Tissue traits that serve as biomarkers of neoplastic change are usually related to tissue scattering properties, the statistical properties of echo or the texture of tumor images and parametric maps. This classification is based on the differences in the values of these parameters between neoplastic and benign lesions that reflect changes in tissue structure caused by tumour development.

Histopathological analysis shows the differences between the tissue of benign and malignant tumours. Malignant tumours are rich in cellularity; the cells tend to form cell clusters. Benign tumours have more regular arrangement of the cells. Additionally, invasive ductal carcinoma, which forms the majority (80–90%) of invasive breast cancers is characterized by a significantly increased amount of dense fibrous tissue stroma^18^. It is important for classification techniques that the specific morphological characteristics of cancer tissue are associated with malignancy and have a significant impact on the tumour image and parameters calculated from its ultrasonic echoes. These features result in more complex scatterer composition in malignant tumours^19^, which can lead to specific signature of backscattered echoes, in QUS.

The scattering properties of tissues can be evaluated by modelling the statistics of the signal envelope using the probability density function (PDF). An overview of distributions used to model scattered signals in soft tissue can be found in the work of Destrempes and Cloutier^20^. One of the most important distributions used for modelling soft tissue scattering is the Nakagami distribution^21^. A good description of tissue scattering statistics and its ease in determining the value of the shape parameter made the distribution of Nakagami very popular in QUS techniques^16,19,22–27^. Another feature studied in the context of tumour classification is the information entropy, which is a measure of uncertainty of a random variable^28,29^. Hughes proposed the use of entropy in quantitative assessment of changes in scattering medium structure^30^. Tsui *et al*. have shown that entropy is sensitive to the variability in concentration of scatterers in tissue phantoms^31^. They then proposed a parametric imaging method based on the entropy determined in small windows^32^. Another example of the QUS parameter is the texture of the ultrasound image, which also reflects the variability in tumour’s structural echogenicity, as it describes the correlation between spatial distribution and the intensity of pixels in the image. The most often used texture-feature parameters are extracted from the gray-level co-occurrence matrix (GLCM). This method was first described by Haralick *et al*.^33^ and now is often used in ultrasound tumour classification^25,34^.

The results of the breast change classification presented so far have been based on data from the inside of the assessed tumours. However, there are reasons to also use data from the tissue surrounding the tumour, as malignant and benign lesions can have different effects on neighboring tissues^18^. They differ in morphological features of the cells and essentially the stromal component proliferation. Malignant tumours are not encapsulated, not cohesive and characterized with irregular pattern of growth. Their borders are not well defined and they spread into adjacent tissue rather than displacing or pushing it aside and cause its damage. On the contrary, benign tumours usually have a covering made up of normal cells and their borders are mostly well-defined. They do not penetrate the adjacent tissue and do not damage it.

Furthermore, the B-mode examination underestimates the size of the tumour. Gruber *et al*.^35^ studied the imaging of tumours in 121 patients using a mammography, sonography and magnetic resonance methods to assess which one is the most accurate in pretherapeutic sizing of primary breast cancer. Tumour size was found to be significantly underestimated in ultrasound, and the mean difference between the sonographic and histological size was 8 mm. It is worth noting that the greatest difference between sonographic sizing and actual histological tumour size was found with invasive lobular breast cancer. These results have been confirmed by Stein *et al*. who analysed data from 6543 breast cancer patients and assessed the accuracy of tumour size measurement by ultrasound^36^. The mean tumour diameter determined by ultrasound was 18.3 mm, whereas the histological mean tumour diameter was 20.8 mm. From these results it follows that the analysis of data limited only to the area of the tumour visible in the B-mode image does not take into account tissue changes in the peripheral area of the tumour and its close vicinity. These areas are important in the classification due to their various modifications during the development of malignant and benign tumours.

The presented study investigated the possibility of distinguishing between malignant and benign tumors using QUS parameters calculated on the basis of signals scattered in the tissue surrounding the tumors, which is the main novelty of the research. The ultrasound data was acquired from 116 patients diagnosed with a suspicious breast lesion. The parameters used in tumour classification were the shape parameter of the Nakagami distribution, the entropy, and ten texture parameters. In our study, parameters determined for the tumour and the rim of the tissue surrounding the tumour were used as independent features in the construction of multi-parametric classifiers.

The observation in this study suggests that the QUS examination of the peritumoral tissue is more effective than the study of the tumour tissue itself. A multi-parametric classifier operating on data from tissue surrounding tumours distinguished changes better than the best classifier operating only on cancer data. On the other hand, the classifier consisting of parameters based separately on tumour and peritumoral tissue data proved to be the best, which suggests the complementarity of quantitative ultrasound information contained in the tumour and external tumour tissue.

## Methods

### Data acquisition

Research was carried out in the Department of Radiology, Maria Skłodowska-Curie Memorial Institute of Oncology in Warsaw. The Institutional Review Board approved the study protocol. All the procedures performed in the study that involved human participants were in accordance with guidelines set by the 1964 WMA Declaration of Helsinki and its later amendments or comparable ethical standards. All patients signed the informed consent for breast US examination and for ‘backscatter US’ statistical studies. Ultrasound B-mode images and radiofrequency data (RF) were acquired by an experienced sonographer using an Ultrasonix SonicTOUCH^®^ machine (Ultrasonix Medical Corporation, Richmond, BC, Canada) equipped with a L14-5/38 linear probe. The transmit frequency was set at 10 MHz (centre frequency ~7.2 MHz), and the sampling frequency was equal to 40 MHz. The focus was set at the middle of tumour and the number of lines per image was 510. The breast US examinations were performed according to the American College of Radiology BI-RADS guidelines using longitudinal and transverse scan planes^4^. Lesions of solid BI-RADS category 3, 4, or 5 were included in this study. Ultrasound data from 116 tumours were collected, including 57 malignant and 59 benign cases. The group of malignant lesions contained following tumor types: ductal (21), lobular (10), cribriform (8), tubular (5), micropapillary (3), and mixed (10). The tumors were assessed as low grade (13), intermediate grade (35) and high grade (9). The tumour contour was determined manually by an experienced breast sonographer. Minimal, mean and maximal tumour equivalent diameter (i.e. diameter of a circle whose area is equal to the area of the tumour) in the data set was equal 4.7, 12.7, and 26.6 mm respectively. Core biopsy samples were taken from patients with BI-RADS 4 and 5 lesions. Patients with BI-RADS 3 underwent the fine needle aspiration biopsy (FNAB) and 2 year follow-up. Ultrasound data were categorized on the basis of histopathological, cytological and patient observation findings that diagnosed benign or malignant tumour.

### ROI selection

**Figure 1:**
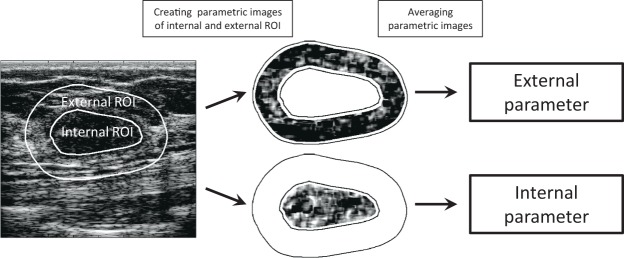
Diagram showing the extraction of internal and external ROI from the B-mode image, parametric images of both ROIs and the ROI-averaged parameters determined from them.

QUS analysis was performed on selected regions of interest (ROI) – a tumour area (internal ROI) and an area surrounding the tumour (external ROI). An example of a tumour image with the ROIs as well as the further data processing scheme is shown in Fig. 1. The width of the analysed tumour rim was selected based on the results of the classification using individual parameters. On average, the best classification results were obtained with a rim width of around 5 mm. This 5 mm width of tissue surrounding the tumours was analysed in all comparisons of the tumour classification efficiency based on data from the internal and external ROI. For convenience, throughout the text, the tissue contained in the contour of the tumour identified by the sonographer is called ‘internal ROI’ and the tissue in the 5 mm thick rim that surrounds the tumour is called ‘external ROI’. Internal parameters define parameters that operate on internal ROI data, as opposed to external parameters that use data from an external ROI. Internal and external classifiers are defined by analogy.

### Nakagami shape parameter

The Nakagami shape parameter was used to characterize tissue microstructure. The probability density function (PDF) of the Nakagami distribution is given by equation (1) ^21^:

1 $$P(A)=\frac{2}{{\rm{\Gamma }}(NAK)}{(\frac{NAK}{{\rm{\Omega }}})}^{NAK}{A}^{2NAK-1}\,\exp \,(-\frac{NAK}{{\rm{\Omega }}}{A}^{2})$$

where ${\rm{\Gamma }}$ is the gamma function, *NAK* is the shape parameter, and ${\rm{\Omega }}$ is the scaling parameter associated with average signal power. The shape parameter *NAK* was estimated using the method of moments^21^ according to the following formula (2):

2 $$NAK=\frac{{\langle {A}^{2}\rangle }^{2}}{{\sigma }^{2}({A}^{2})}$$

where *A* is the amplitude, $\langle \rangle$ is the mean and ${\sigma }^{2}()$ is the variance.

### Entropy

In this study, weighted entropy^37^ was used as a measure of local signal envelope heterogeneity. Weighted entropy (*ENT*) was estimated using formula (3):

3 $$ENT(A)=-\,\sum _{i=1}^{n}\,w({A}_{i})P({A}_{i}){lo}{{g}}_{2}P({A}_{i})$$

where *n* represented the number of samples in a data block, and *w* and *P* were the weight and the probability associated with the *i*-th amplitude value *A*_*i*_, respectively. Amplitude values *A* divided by the sum of all amplitude values in the window were used as the weights. The probability *P* was estimated using the histogram method.

### Textural features

The textural features of the US images were extracted using the Gray Level Co-occurrence Matrix (GLCM). The GLCM is a matrix that contains probabilities of occurrence of certain gray tones in a pair of pixels being in a particular relative spatial position. For the purpose of the GLCM calculation the gray scale of the ultrasound images was limited to a 20–100 dB range and quantized into 20 gray levels which resulted in GLCMs of size 20 × 20. The spatial relationship in any considered pair of pixels was defined as a vertical or horizontal displacement by 0.3 mm (4 pixels). We considered the vertical and horizontal textural features to carry different information, and therefore the GLCMs obtained for vertical and horizontal spatial relationships were used for determination of separate texture parameters marked with the letters ‘V’ and ‘H’ respectively. This approach is different in comparison to the previously cited works^25,34^, where the average parameters were used, estimated on the basis of the GLCM matrix calculated in four directions (0°, 45°, 90° and 135°). Based on each GLCM a number of texture parameters were calculated. These were: contrast (*CON*), correlation (*COR*), energy (*ENE*), homogeneity (*HOM*), and variance (*VAR*):

4 $$CON=\sum _{i,j}\,|i-j{|}^{2}GLCM(i,j)$$

5 $$COR=\sum _{i,j}\,\frac{(i-{\mu }_{i})\,(j-{\mu }_{j})}{{\sigma }_{i}{\sigma }_{j}}GLCM(i,j)$$

6 $$ENE=\sum _{i,j}\,GLCM{(i,j)}^{2}$$

7 $$HOM=\sum _{i,j}\,\frac{GLCM(i,j)}{1+|i-j{|}^{2}}$$

8 $$VAR=\sum _{i,j}\,{(i-{\mu }_{i})}^{2}GLCM(i,j)$$

where *i* and *j* indicated the discrete gray levels and were also the indices of GLCM elements. The *μ* and *σ* denoted the mean and standard deviation of the *i* and *j* coordinates. It must be noted that each GLCM was normalized (so that sum of its elements was equal 1) before determination of said parameters.

### Parameters estimation

The spatial parametric images of tumours and the surrounding rims were generated using the sliding window technique. The parametric image represented a map of the parameter value distribution in the analysed ROI, external or internal. The parameter value (Nakagami parameter, entropy, or any of the parameters of the texture) was estimated based on the block of ultrasonic data from each window. The window size was equal 1 *mm* × 1 *mm*, which corresponded to three times the pulse length, as recommended by Tsui *et al*.^38^. Window overlap in the axial and lateral direction equaled 92%. Parametric maps were corrected to minimize the impact of acoustic beam formation and the system transfer function. Data from the reference tissue phantom (Dansk Fantom Service, model 1126-B) was obtained with the same scan settings as in the patient study. This data was used to generate reference parametric maps. Based on these maps, correction curves were determined for each of the considered parameters, which were then used to improve the parametric maps of tumors and their surroundings. Next, for each tumour the parameter value was averaged over all pixels of parametric maps in the two scanned planes of tumour. The averaged parameter was used as a predictor of neoplastic changes in subsequent classifications. The data processing scheme is presented in Fig. 1.

### Statistical analysis

Twelve parameters were considered including Nakagami parameter, weighted entropy, and ten texture parameters determined from the GLCM matrix, five for each vertical and horizontal spatial relationship. For the classification of tumours based on single-parameter and multi-parameter classifiers, the k-nearest neighbours (k-NN) algorithm^39^ was used, with k equal to 4 and standardized Euclidean distance. Classifiers were cross-validated through the ‘leave-one-out’ technique^40^. Evaluation of the classification results was based on the analysis of the receiver operating characteristic (ROC) curves^41^, in particular the area under the ROC curve (AUC), sensitivity, specificity, and accuracy. In order to compare the effectiveness of the classification based on internal and external ROI data, AUC values were determined for all single-parameter classifiers (SPCs) and multi-parameter classifiers (MPCs) built up of all the possible combinations of single parameters. This ‘exhaustive search’ approach^42^ was applied to three types of MPCs. The first one consisted of parameters determined using data from the tumour (internal ROI), the second from data collected from the tumour rim (external ROI), and the third one used both types of parameters. The best classifiers of each type were chosen based on their highest AUC values. Parameters included in selected MPCs are shown in the results section. To assess the statistical significance of classification results, corresponding p-values were determined through a two-sided Wilcoxon rank sum test. All calculations were done using Matlab^®^ 2017a (The MathWorks, Inc., Natick, MA).

## Results

**Figure 2:**
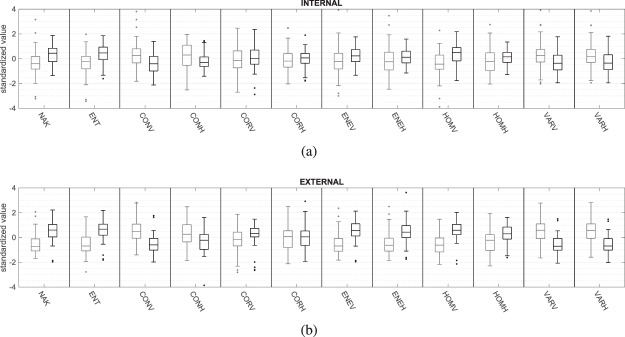
Comparison of standardized values of individual parameters, internal (**a**) and external (**b**), calculated for benign (gray lines) and malignant (black lines) tumours.

**Figure 3:**
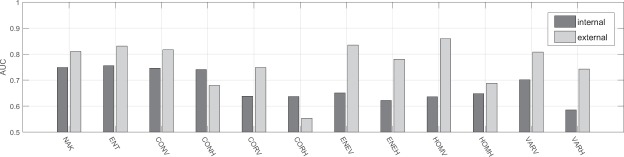
AUC values comparison between single-parametric classifiers estimated inside and outside the tumour.

**Figure 4:**
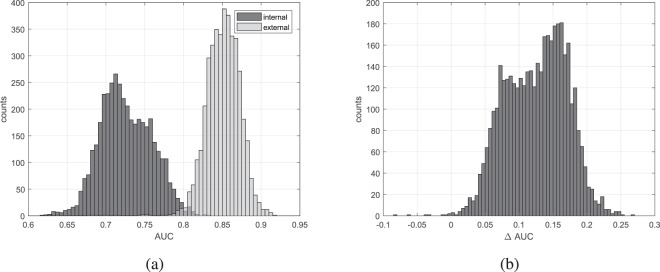
AUC values comparison of all internal and external multi-parametric classifiers (**a**) and AUC differences between corresponding external and internal multi-parametric classifiers (**b**).

Boxplots characterizing individual parameters values estimated from benign and malignant tumours are presented in Fig. 2, in gray and black respectively. The maximum whiskers length was specified as a 1.5 interquartile range. The AUC values for all single-parametric classifiers estimated inside and outside the tumour are shown in Fig. 3. The usefulness of the tissue surrounding breast tumours to classify benign and malignant lesions was examined using multi-parametric classifiers. For this purpose, the efficiency of classification of all possible classifiers built based on the internal parameters themselves and only external parameters was checked. As a result, two groups of AUC values were obtained, each with a count of 4095. A comparison of the AUC values in these two groups is shown in Fig. 4a. Figure 4b shows a histogram of the differences between the AUC values estimated for the same classifier but first using data collected outside the tumour (external ROI) and the second time operating on the data from the tumour tissue (internal ROI).

The AUC, sensitivity, specificity, accuracy, and statistical significance of SPCs and the best (regarding AUC values) MPCs are presented in the Tables 1 and 2 respectively. Statistical significance has been divided into three classes; extremely significant ($p < 0.001$), highly significant ($p < 0.01$) and significant ($p < 0.05$) and marked with ***, **, and *, respectively. Table 2 contains performance assessment of the best MPC built only from internal parameters, the best MPC based only on external parameters, and the best MPC using the external and internal parameters together. The constituent parameters of the best classifiers were as follows. Parameters *NAK*, *CONH*, *ENEV*, *HOMV*, and *VARH* were components of the best internal classifier. Parameters *CORV*, *CORH*, *HOMV*, *VARV*, and *VARH* were components of the best external classifier. The combined multi-parametric classifier used the parameters *CORV* and *ENEV* determined from the tumour and *CONV*, *ENEV*, *ENEH*, *HOMV*, and *HOMH* determined on the basis of data from the tissue surrounding the tumour.

**Table 1:** Performance comparison of single-parametric internal and external classifiers.

ROI	Parameter used	AUC	Sensitivity	Specificity	Accuracy	Statistical significance
Internal	NAK	0.75	0.72	0.81	0.77	***
	ENT	0.76	0.82	0.69	0.76	***
	CONV	0.75	0.70	0.81	0.76	***
	CONH	0.74	0.70	0.80	0.75	***
	CORV	0.64	0.67	0.75	0.71	**
	CORH	0.64	0.72	0.68	0.70	**
	ENEV	0.65	0.67	0.69	0.68	**
	ENEH	0.62	0.58	0.76	0.67	*
	HOMV	0.64	0.61	0.73	0.67	**
	HOMH	0.65	0.82	0.58	0.70	**
	VARV	0.70	0.47	0.93	0.71	***
	VARH	0.59	0.53	0.81	0.67	non-significant
External	NAK	0.81	0.98	0.61	0.79	***
	ENT	0.83	0.72	0.92	0.82	***
	CONV	0.82	0.81	0.81	0.81	***
	CONH	0.68	0.95	0.46	0.70	***
	CORV	0.75	0.79	0.73	0.76	***
	CORH	0.55	0.28	0.93	0.61	non-significant
	ENEV	0.83	0.84	0.76	0.80	***
	ENEH	0.78	0.82	0.73	0.78	***
	HOMV	0.86	0.72	0.93	0.83	***
	HOMH	0.69	0.70	0.73	0.72	***
	VARV	0.81	0.82	0.81	0.82	***
	VARH	0.74	0.82	0.68	0.75	***

Statistical significance is divided into classes based on p-values and marked as follows: non-significant ($p\ge 0.05$), *($p < 0.05$), **($p < 0.01$) and ***($p < 0.001$).

**Table 2:** The performance parameters of the best multi-parametric classifiers.

Parameters used	AUC	Sensitivity	Specificity	Accuracy	Statistical significance
Internal	0.83	0.68	0.95	0.82	***
External	0.92	0.81	0.97	0.89	***
Internal & External	0.94	0.93	0.88	0.91	***

Three asterisks indicate very high statistical significance ($p < 0.001$).

## Discussion

In the presented studies, the effectiveness of breast cancer classification was compared on the basis of QUS analysis of two types of ultrasound data, collected from within the tumour and from the surrounding tissue. The quantitative parameters included the shape parameter of the Nakagami distribution, weighted entropy, and a set of texture parameters. In Fig. 2 boxplot pairs corresponding to the values of parameters determined for benign and malignant tumours are shown. For almost all boxplot pairs, the difference of medians in the external parameter group was greater than in the group of internal parameters. The only exceptions were *CONH* and *CORH*. The greater difference between medians generally translates into a higher classification efficiency. This is clearly visible in Fig. 3, which compares the AUC values for lesions classification using external and internal SPCs. In most cases the AUC for external parameters was larger than for internal parameters. The only exceptions were again *CONH* and *CORH* parameters. The largest difference in AUC was observed for the *HOMV* parameter (ΔAUC = 0.22). The AUC values for external SPCs in six cases reached values above 0.8. *P*-values for SPCs indicated that most of them are statistically significant from the point of view of differentiation of benign and malignant tumours. Among external SPCs, 11 out of 12 were characterized by a very high statistical significance ($p < 0.001$). In the case of internal SPCs, there were five cases with similar statistical significance. No statistically significant difference ($p > 0.05$) was found for the two SPCs, based on internal *VARH* and external *CORH*, and the resulting AUC values (0.59 and 0.55 respectively) were close to 0.5 which corresponds to a random classification. It is worth noting that both of these parameters were calculated from the GLCM matrix determined for the horizontal direction. Their counterparts counted for vertical direction GLCM were characterized by a significantly lower p-value and higher AUC (see Table 1). The reason for this difference can be twofold. The Point Spread Function (PSF) of ultrasonic imaging systems operating in the pulse-echo mode is diametrically different for vertical and horizontal directions, which can translate into the estimates of texture parameters determined horizontally and vertically. Another explanation may be that malignant tumours tend to grow deeper into the breast more often than benign, which may cause their non-isotropic structure. It seems that the textural parameters of ultrasound images for vertical and horizontal directions should be considered as describing different texture properties. Their mixing, i.e. the calculation of the average parameter values for several directions at once, may lead to the loss of information contained in each of them individually. However, it should be emphasized that the proposed explanations are only hypothetical and require further research.

Histograms of AUC values calculated for multi-parametric classifiers are shown in Fig. 4a. It can be seen that the external MPCs on average work better compared to internal ones. Differences were also determined between the AUC values estimated in external and internal ROI for each MPC. The histogram of the ΔAUC is shown in Fig. 4b. It is very clearly seen that most of the differences are positive, which means that in most cases the external classifier had a higher AUC compared to its internal equivalent. For 4095 compared classifiers, only eight internal classifiers had a higher AUC (Fig. 4b). As in the case of single-parameter classifiers, multi-parametric classifiers using external parameters gave clearly better results than those using internal parameters. It is also worth noting that the effectiveness of the best classifier operating solely on the basis of external parameters was similar to the performance of the best classifier (Table 1), which used two internal parameters and five external parameters and reached the highest AUC (0.94) and accuracy (0.91).

The presented results suggest difference in the ultrasound backscatter from the tissue surrounding the benign and malignant tumors. These results are consistent with previous studies which have provided a potential biological explanation. The peritumoral tissue had been intensively studied due to its important role in the development of cancer and its spread. Peritumoral invasion of cancer cells is a prognostic factor significantly associated with an increased risk of recurrence and death in patients with breast cancer^43,44^. The alterations in stroma play an important role in invasiveness of the breast cancer. Itoh *et al*.^45^ suggested that changes in the peritumoral stroma, in particular an increase in stiffness was due to infiltration of tumor cells. In the early stage of cancer spread, the amount of collagen in the tumor’s surrounding tissue increases^46^. These collagen changes on the margins of the tumour are known as ‘desmoplasia’. In breast cancer it is increased collagen cross-linking which leads to increased focal adhesion and induces the tumour invasion^47^. What’s more, the actual orientation of collagen fibers also facilitates the invasion of cancer cells^48^. The fibers, which are ‘curly’ and anisotropic in normal stroma, in the case of invasive cancer form a characteristic signature of straight and bundled collagen fibers oriented perpendicularly to the tumor border^46,49,50^. Tumor cells on the border of the tumor with the stroma enter the stroma along the radially arranged collagen fibers. It has been suggested that these tumor-associated collagen signatures may serve as a feature helpful in identifying and characterizing breast tumors^50^. An important role of the peritumoral tissue was demonstrated by Tadayyon *et al*.^51^ when monitoring the effects of neoadjuvant chemotherapy. Changes in the tissue surrounding the tumor caused by the therapy were sufficient to detect them using ultrasound. The QUS parameters estimated in the tumor and tumor margin indicated the degree of tumor response to therapy and could predict a 5 year survival without recurrence. A high diagnostic value of acoustic features of peritumoral tissue has been suggested, which is consistent with our results. It is also worth noting that Tadayyon *et al*. has chosen a margin width of 5 mm as optimal for characterizing the tumor response to chemotherapy, and the same width of the tumor margin was optimal in the presented above classification results. This suggests the existence of a certain range of the thickness of the peritumoral tissue, in which malignant tumors affect the acoustic properties of the tissue and changes in these properties can be estimated using the QUS methods.

The use of external parameters improves the classification, but also has some limitations. Some tumors are located so shallow that the surrounding tissue includes the skin, which affects the determined QUS parameters. In such cases, it is not recommended to use part of the skin-related QUS map when determining the average parameters. Incorrect determination of external parameters may be also caused by high attenuation in the tumor. In the resulting acoustic shadow the signal-to-noise ratio (SNR) is low and the QUS parameters are subject to a large error. In extreme cases, the acoustic shadow makes it impossible to determine the lower edge of the tumor, thus making it impossible to determine areas for calculating internal and external parameters.

## Conclusions

The obtained results indicate that the signals received from the surroundings of breast tumours contain important information allowing for the classification of tumours as malignant or benign. Moreover, the majority of the individual parameters tested showed a higher classification efficiency when they were calculated for the tumour surroundings, and not for its interior. A similar relationship was observed in the case of multi-parametric classifiers, which is important in the context of the development of new cancer classification methods based on QUS techniques. In our research, the multi-parameter classifier, using a combination of internal and external parameters, was the best, although the best classifier using only external parameters was not significantly worse. It also suggests that external parameters are even more important and more valuable for classification than internal ones. In conclusion, we believe that quantitative analysis of peritumoral tissue can complement conventional US of breast tumours, thereby making it easier to diagnose breast lesions. The results of the study also show that alterations in peritumoral stroma caused by malignant neoplasms translate into changes in tissue properties that can be detected using ultrasonic quantitative techniques. These findings may be useful in research on the development and spread of cancerous tissue.

## Data Availability

The datasets generated during the current study are stored and available in Department of Ultrasound of Institute of Fundamental Technological Research (IFTR, Warsaw, Poland) on reasonable request.

## References

[1] Ferlay, J. *et al*. Cancer incidence and mortality worldwide: sources, methods and major patterns in globocan 2012. *International journal of cancer* **136**, 10.1002/ijc.29210 (2015). 10.1002/ijc.29210 25220842

[2] Duffy SW (2002). The impact of organized mammography service screening on breast carcinoma mortality in seven swedish counties. Cancer.

[3] Kolb TM, Lichy J, Newhouse JH (2002). Comparison of the performance of screening mammography, physical examination, and breast us and evaluation of factors that influence them: an analysis of 27,825 patient evaluations. Radiology.

[4] Mendelson, E. *et al*. Acr bi-rads® ultrasound. *ACR BI-RADS Atlas*, *Breast Imaging Reporting and Data System*. *Reston*, *VA*, *American College of Radiology* **149** (2013).

[5] Shoma A, Moutamed A, Ameen M, Abdelwahab A (2006). Ultrasound for accurate measurement of invasive breast cancer tumor size. The breast journal.

[6] Förnvik D (2010). Breast tomosynthesis: accuracy of tumor measurement compared with digital mammography and ultrasonography. Acta radiologica.

[7] Berg WA, Blume JD, Cormack JB, Mendelson EB (2012). Training the acrin 6666 investigators and effects of feedback on breast ultrasound interpretive performance and agreement in bi-rads ultrasound feature analysis. American Journal of Roentgenology.

[8] Brem RF, Lenihan MJ, Lieberman J, Torrente J (2015). Screening breast ultrasound: past, present, and future. American Journal of Roentgenology.

[9] Abdel-Nasser M, Melendez J, Moreno A, Omer OA, Puig D (2017). Breast tumor classification in ultrasound images using texture analysis and super-resolution methods. Engineering Applications of Artificial Intelligence.

[10] Venkatesh SS, Levenback BJ, Sultan LR, Bouzghar G, Sehgal CM (2015). Going beyond a first reader: A machine learning methodology for optimizing cost and performance in breast ultrasound diagnosis. Ultrasound in medicine & biology.

[11] Wu W-J, Lin S-W, Moon WK (2015). An artificial immune system-based support vector machine approach for classifying ultrasound breast tumor images. Journal of digital imaging.

[12] Hizukuri A, Nakayama R (2018). Computer-aided diagnosis scheme for determining histological classification of breast lesions on ultrasonographic images using convolutional neural network. Diagnostics.

[13] Chen C-M (2003). Breast lesions on sonograms: computer-aided diagnosis with nearly setting-independent features and artificial neural networks. Radiology.

[14] Yassin NI, Omran S, El Houby EM, Allam H (2018). Machine learning techniques for breast cancer computer aided diagnosis using different image modalities: A systematic review. Computer methods and programs in biomedicine.

[15] Nam K, Zagzebski JA, Hall TJ (2013). Quantitative assessment of *in vivo* breast masses using ultrasound attenuation and backscatter. Ultrasonic imaging.

[16] Dobruch-Sobczak K, Piotrzkowska-Wróblewska H, Roszkowska-Purska K, Nowicki A, Jakubowski W (2017). Usefulness of combined bi-rads analysis and nakagami statistics of ultrasound echoes in the diagnosis of breast lesions. Clinical radiology.

[17] Trop I (2014). The added value of statistical modeling of backscatter properties in the management of breast lesions at us. Radiology.

[18] Lakhani, S. R. *WHO Classification of Tumours of the Breast* (International Agency for Research on Cancer, 2012).

[19] Tsui PH (2010). Ultrasonic nakagami imaging: A strategy to visualize the scatterer properties of benign and malignant breast tumors. Ultrasound in medicine & biology.

[20] Destrempes F, Cloutier G (2010). A critical review and uniformized representation of statistical distributions modeling the ultrasound echo envelope. Ultrasound in medicine & biology.

[21] Nakagami M (1960). The m-distribution-a general formula of intensity distribution of rapid fading. Statistical Method of Radio Propagation.

[22] Shankar PM (2000). A general statistical model for ultrasonic backscattering from tissues. IEEE transactions on ultrasonics, ferroelectrics, and frequency control.

[23] Shankar PM (2001). Classification of ultrasonic b-mode images of breast masses using nakagami distribution. IEEE transactions on ultrasonics, ferroelectrics, and frequency control.

[24] Tsui PH (2009). Use of nakagami statistics and empirical mode decomposition for ultrasound tissue characterization by a nonfocused transducer. Ultrasound in medicine & biology.

[25] Liao YY (2011). Classification of scattering media within benign and malignant breast tumors based on ultrasound texture-feature-based and nakagami-parameter images. Medical physics.

[26] Ma HY (2016). Ultrasound window-modulated compounding nakagami imaging: Resolution improvement and computational acceleration for liver characterization. Ultrasonics.

[27] Tsui PH, Wan YL (2016). Application of ultrasound nakagami imaging for the diagnosis of fatty liver. Journal of Medical Ultrasound.

[28] Shannon CE (1948). A mathematical theory of communication. The Bell System Technical Journal.

[29] Wang QA (2008). Probability distribution and entropy as a measure of uncertainty. Journal of Physics A: Mathematical and Theoretical.

[30] Hughes MS (1993). Analysis of digitized waveforms using shannon entropy. The Journal of the Acoustical Society of America.

[31] Tsui PH (2015). Ultrasound detection of scatterer concentration by weighted entropy. Entropy.

[32] Tsui, P. H. *et al*. Small-window parametric imaging based on information entropy for ultrasound tissue characterization. *Scientific reports* **7**, 10.1038/srep41004 (2017). 10.1038/srep41004 PMC5247684 28106118

[33] Haralick, R. M., Shanmugam, K. & Dinstein, I. Textural features for image classification. *IEEE Transactions on systems*, *man*, *and cybernetics* 610–621, 10.1109/TSMC.1973.4309314 (1973).

[34] Sadeghi-Naini A (2017). Breast-lesion characterization using textural features of quantitative ultrasound parametric maps. Scientific reports.

[35] Gruber IV (2013). Measurement of tumour size with mammography, sonography and magnetic resonance imaging as compared to histological tumour size in primary breast cancer. BMC cancer.

[36] Stein RG (2016). The impact of breast cancer biological subtyping on tumor size assessment by ultrasound and mammography-a retrospective multicenter cohort study of 6543 primary breast cancer patients. BMC cancer.

[37] Guiaşu S (1971). Weighted entropy. Reports on Mathematical Physics.

[38] Tsui PH, Ma HY, Zhou Z, Ho MC, Lee YH (2014). Window-modulated compounding nakagami imaging for ultrasound tissue characterization. Ultrasonics.

[39] Cover T, Hart P (1967). Nearest neighbor pattern classification. IEEE transactions on information theory.

[40] Hastie, T., Tibshirani, R. & Friedman, J. *The elements of statistical learning: data mining*, *inference*, *and prediction* (Springer series in statistics, 2009).

[41] Fawcett T (2006). An introduction to roc analysis. Pattern Recognition Letters.

[42] Guyon, I., Gunn, S., Nikravesh, M. & Zadeh, L. A. *Feature Extraction*, *Foundations and Applications*, 10.1007/978-3-540-35488-8 (Springer, Berlin, Heidelberg, 2006).

[43] Colleoni M (2007). Prognostic role of the extent of peritumoral vascular invasion in operable breast cancer. Annals of oncology.

[44] De Mascarel I (1998). Obvious peritumoral emboli: an elusive prognostic factor reappraised. multivariate analysis of 1320 node-negative breast cancers. European journal of cancer.

[45] Itoh A (2006). Breast disease: clinical application of us elastography for diagnosis. Radiology.

[46] Conklin MW (2011). Aligned collagen is a prognostic signature for survival in human breast carcinoma. The American journal of pathology.

[47] Levental KR (2009). Matrix crosslinking forces tumor progression by enhancing integrin signaling. Cell.

[48] Conklin MW, Keely PJ (2012). Why the stroma matters in breast cancer: insights into breast cancer patient outcomes through the examination of stromal biomarkers. Cell adhesion & migration.

[49] Egeblad M, Rasch MG, Weaver VM (2010). Dynamic interplay between the collagen scaffold and tumor evolution. Current opinion in cell biology.

[50] Provenzano PP (2006). Collagen reorganization at the tumor-stromal interface facilitates local invasion. BMC medicine.

[51] Tadayyon H (2017). A priori prediction of neoadjuvant chemotherapy response and survival in breast cancer patients using quantitative ultrasound. Scientific reports.

